# Challenging treatment of severe hypotension following tracheal intubation in a patient with primary hyperparathyroidism: A case report

**DOI:** 10.1097/MD.0000000000039510

**Published:** 2024-08-30

**Authors:** Yuhei Koyama, Shinsuke Hamaguchi

**Affiliations:** a Department of Anesthesiology and Pain Medicine, Dokkyo Medical University School of Medicine, Tochigi, Japan.

**Keywords:** case report, general anesthesia, hypotension, primary hyperparathyroidism, tachycardia

## Abstract

**Rationale:**

Primary hyperparathyroidism (PHPT), which is characterized by increased parathyroid hormone secretion, typically manifests as hypercalcemia and hypertension. Here, we report a case of severe hypotension following tracheal intubation during anesthesia induction in a patient with PHPT, in contrast to the expected hypertensive response.

**Patient concerns:**

A 52-year-old man presented with nausea after eating, leg pain when walking, and headaches.

**Diagnosis:**

Based on the blood test and computed tomography results, he was diagnosed with PHPT.

**Interventions:**

The patient underwent parathyroidectomy under general anesthesia. After induction anesthesia and tracheal intubation, severe acute hypotension and tachycardia suddenly developed. To treat hypotensive shock, we immediately administered ephedrine and phenylephrine and infused Ringer solution.

**Outcomes:**

The symptoms of hypotensive shock were alleviated by this intervention.

**Lessons:**

We speculate that the cause of his severe hypotension was vasodilation due to the transient release of parathyroid hormone from mechanical stimulation by anesthetic procedures, such as tracheal intubation, combined with hypercalcemia-induced severe dehydration. Moreover, we speculate that fluid resuscitation stabilized his condition and helped achieve a successful surgical outcome. The possibility of severe hypotension after anesthesia induction should be anticipated, and management of cases with severe dehydration should be optimized during the anesthetic management of patients with PHPT.

## 1. Introduction

Primary hyperparathyroidism (PHPT), caused by parathyroid adenoma in 84% and parathyroid hyperplasia in 15% of cases, is an endocrine disorder that induces metabolic dysregulation of calcium and phosphate owing to increased production and secretion of parathyroid hormone (PTH).^[1]^ Typically, hypercalcemia in patients with PHPT leads to hypertension via systemic vascular contraction resulting from increased serum calcium levels.^[2]^ However, we encountered a challenging case in which a patient with PHPT experienced severe hypotension after tracheal intubation. Therefore, we report our experiences with this case of severe hypotension caused by PHPT after obtaining the patient’s consent. Our findings highlight the importance of considering that general anesthesia in patients with PHPT can cause severe hypotension and hypertension during anesthesia induction, thus emphasizing the need to correct preoperative dehydration in these patients adequately. This case report follows the CARE Guidelines.^[3]^

## 2. Narrative

A 52-year-old man (height 171.5 cm, weight 63.8 kg), with unremarkable medical and family history, presented with nausea after eating, leg pain when walking, and headaches that had started 8 months previously. The patient was referred to the Department of Endocrinology and Metabolism for further examination and management. Preoperative vital signs were within the normal range: blood pressure, 110 to 140/70 to 90 mm Hg; pulse, 60 to 100/min. Body temperature 36.4 °C, and respiratory rate, 16 breaths/min. Preoperative blood tests revealed an abnormally elevated intact PTH (2611.0 pg/mL, normal range: 18.5–88.0 pg/mL) and serum calcium (15.6 mg/dL, normal range: 8.8–10.1 mg/dL) levels, whereas the serum albumin level was 3.9 g/dL (normal range: 4.1–5.1 g/dL). Chest radiography and electrocardiography revealed no abnormalities. The QT interval on an electrocardiogram was 0.357 seconds, and the QTc interval was 0.416/0.395 seconds, with no prolongation or shortening. Cervical computed tomography revealed a solid tumor with a diameter of 2 cm in the left inferior parathyroid artery. The patient received 6 mg/day of evocalcet, 17.5 mg/day of risedronate for hypercalcemia, and 2 g/day of magnesium oxide for constipation. Following these assessments, the patient was diagnosed with a left inferior functional parathyroid adenoma, for which a parathyroidectomy was planned at the Otorhinolaryngology Department of our hospital. The results of the blood biochemical tests performed 11 weeks before surgery were as follows: total protein 6.9 g/dL, albumin 3.5 g/dL, urea nitrogen 16.5 mg/dL, and sodium 141 mmol/L. Potassium 3.5 mmol/dL, chloride 101 mmol/dL, calcium 11.6 mg/dL, creatinine 1.02 mg/dL, red blood cell count 3.71 1012/L, hemoglobin 11.1 g/dL, and hematocrit 33.5%. Subsequently, the results of blood biochemical tests conducted 3 weeks before surgery were as follows: total protein 7.2 g/dL, albumin 3.9 g/dL, urea nitrogen 17.8 mg/dL, and sodium 136 mmol/L. Potassium 3.1 mmol/L, chloride 101 mmol/L, calcium 15.6 mg/dL, creatinine 1.91 mg/dL, red blood cell count 4.30 1012/L, hemoglobin 12.7 g/dL, and hematocrit 38.0%. We planned anesthetic management for this procedure based on the results of the preoperative examination.

**Figure 1.**
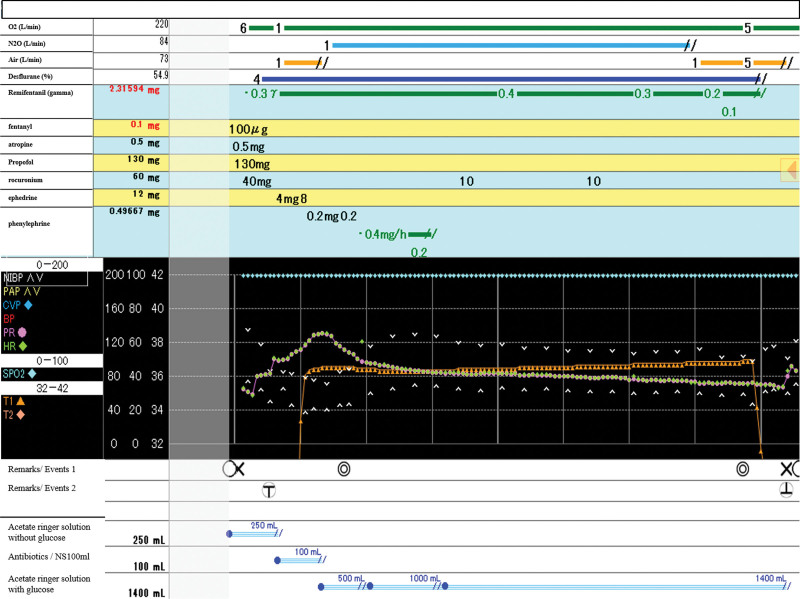
Anesthesia record. ◯ Patient entering and leaving the room. × Anesthesia start/end. ○ T Intubation. ○⊥ Extubation. ◎ Operation start/end.

The patient was admitted to the hospital 1 day before surgery. An anesthetic chart of this case is shown in Figure 1. The patient was prepared with an intravenous line on his left forearm and 250 mL of Ringer acetate solution upon entering the room. We commenced intraoperative monitoring, including electrocardiography, noninvasive blood pressure measurements, and pulse oximetry, as well as preoxygenation with 6 L/min of oxygen and continuous administration of remifentanil (0.3 mg/kg/min). Subsequently, fentanyl (100 μg), atropine (0.5 mg), and propofol (130 mg) were administered, followed by 4% desflurane inhalation after confirming sleep onset. After administering rocuronium (40 mg) for muscle relaxation, the patient was orally intubated with a spiral tracheal tube (internal diameter, 8.0 mm) using a video laryngoscope. Immediately after intubation, the patient’s blood pressure decreased to 84/52 mm Hg, with a gradual increase in the heart rate. Ephedrine (4–8 mg) and phenylephrine (0.2 mg) were intermittently administered but had a limited effect on raising the patient’s blood pressure, which declined to 60/40 mm Hg, while the heart rate increased to 130 bpm. Following infusion of 500 mL of 1% glucose-containing acetate Ringer solution, the blood pressure gradually increased, and the heart rate decreased to <100 bpm. Continued fluid resuscitation using this solution eventually increased the patient’s blood pressure to 120/80 mm Hg, prompting the cessation of continuous phenylephrine administration. Subsequently, no hypotension or tachycardia was observed, and surgery was completed without further complications. The surgery lasted 91 minutes, with an anesthesia time of 125 minutes. Intraoperatively, urine and blood losses were 200 and 60 mL, respectively, with total crystalloid fluid administration of 1750 mL. The patient’s general condition and vital signs remained stable on the day after surgery, with no remarkable events occurring within the admission period. The pathological diagnosis of the tumor was functional parathyroid carcinoma.

## 3. Discussion

In this report, we present our experience with a patient with PHPT who developed severe hypotension after tracheal intubation, which proved challenging to manage despite the known increase in catecholamine responsiveness caused by increased blood renin and angiotensin II levels.^[4,5]^ Our patient developed severe hypotension immediately following tracheal induction; however, vasopressor administration did not result in a rapid increase in blood pressure. To the best of our knowledge, no previous cases of severe hypotension following anesthesia induction in patients with PHPT have been reported.

We hypothesized that hypotension in this patient resulted from a transient increase in PTH release due to stimulation during intubation compounded by chronic hypercalcemia-induced dehydration.

Typically, patients with PHPT present with significantly higher blood PTH levels than healthy individuals. However, Cinamon et al^[6]^ reported significantly higher blood PTH levels before intubation owing to general anesthesia- and tracheal intubation-induced stress. Therefore, increased PTH release in PHPT presumably causes hypertension, as indicated in previous reports.^[3–5]^ In contrast, rapid intravenous injection of PTH also reportedly causes systemic hypotension. In their experimental study, Baksi^[7]^ observed decreased blood pressure in rats administered a rapid intravenous injection of teriparatide, a recombinant human PTH (1-34), and proposed that this decrease may be attributed to the vasodilator effect of PTH itself. In their clinical study, McCarron et al^[8]^ reported a transient decrease in blood pressure in healthy young and older participants after a 2-minute intravenous infusion of human PTH (1-34). Therefore, we speculate that the increased PTH levels, triggered by stimulation during intubation, may have caused severe hypotension in our case owing to its vasodilatory effect and the vasodilatory and inhibitory cardio-constrictive effects of the anesthetics.

Prolonged preoperative water deprivation may further exacerbate fluid volume insufficiency, contributing to hypotension in the present case.

In addition, hypercalcemia, a common complication of PHPT, often leads to severe dehydration, which must be corrected during general anesthesia.^[9]^ Kong et al^[10]^ highlighted the necessity of life-saving infusion therapy in managing parathyroid crises during anesthesia administration. Thus, infusion therapy may prove vital during anesthesia management in such cases. Based on these findings, we inferred that the patient in our case was chronically dehydrated because of severe hypercalcemia. In our case, the values of total protein, albumin, red blood cells, hemoglobin, and hematocrit were elevated in the test values 3 days before surgery compared to the test values 11 days before surgery. Therefore, we should be aware of the potential for dehydration.

Typically, isotonic saline at 200 to 300 mL/h with a target urine output of 100 to 150 mL/h is recommended for replacement fluid therapy in patients with hypercalcemia.^[11]^ In addition, prolonged preoperative water deprivation may further exacerbate fluid volume insufficiency, contributing to hypotension in our case.

## 4. Conclusion

In conclusion, our case report highlights the importance of addressing preoperative dehydration in patients with PHPT. Additionally, we recommend that healthcare providers should be vigilant regarding the risk of severe hypotension and hypertension during anesthesia induction in these patients.

## Author contributions

**Conceptualization:** Shinsuke Hamaguchi.

**Data curation:** Yuhei Koyama, Shinsuke Hamaguchi.

**Investigation:** Yuhei Koyama, Shinsuke Hamaguchi.

**Methodology:** Yuhei Koyama, Shinsuke Hamaguchi.

**Resources:** Yuhei Koyama, Shinsuke Hamaguchi.

**Validation:** Yuhei Koyama, Shinsuke Hamaguchi.

**Writing – original draft:** Yuhei Koyama, Shinsuke Hamaguchi.

**Writing – review & editing:** Shinsuke Hamaguchi.
